# Hypertension among adults in Bangladesh: evidence from a national cross-sectional survey

**DOI:** 10.1186/s12872-016-0197-3

**Published:** 2016-01-25

**Authors:** Muhammad Abdul Baker Chowdhury, Md. Jamal Uddin, Md. Rabiul Haque, Boubakari Ibrahimou

**Affiliations:** Department of Biostatistics, Robert Stempel College of Public Health & Social Work, Florida International University, Miami, USA; Department of Statistics, Shahjalal University of Science and Technology, Sylhet, Bangladesh; Department of Population Sciences, University of Dhaka, Dhaka, Bangladesh

**Keywords:** Hypertension, Non-communicable diseases, Body mass index (BMI), National survey, BDHS, Diabetes, Bangladesh

## Abstract

**Background:**

Hypertension is an increasing problem in Southeast Asia, particularly in Bangladesh. Although some epidemiological studies on hypertension have been conducted in Bangladesh, the factors associated with hypertension in this nation remain unclear. We aimed to determine the factors associated with hypertension among the adults in Bangladesh.

**Methods:**

We conducted a cross-sectional study using data from the nationally representative 2011 Bangladesh Demographic and Health Survey (BDHS). A total of 7,839 (3,964 women and 3,875 men) adults aged 35 years and older who participated in the survey was included. Hypertension was defined by a systolic blood pressure ≥ 140 mmHg and/or, diastolic blood pressure ≥ 90 mmHg and/or, receipt of an anti-hypertensive medication at time of the survey. The degree of association between the risk factors and the outcome was assessed by the odd ratio (OR) obtained from the bivariate and multivariable logistic regression models.

**Results:**

The overall prevalence of hypertension was 26.4 %, and the prevalence was higher in women (32.4 %) than men (20.3 %). Study participants with the age group of 60–69 years had higher odds of having hypertension (AOR: 3.77, 95 % CI: 3.01–4.72) than the age group 35–39 years. Moreover, individuals who had higher educational attainment (AOR: 1.63, 95 % C.I: 1.25–2.14) and higher wealth status (AOR = 1.91, 95 % CI: 1.54–2.38) had higher odds of having hypertension than the individuals with no education and lower social status, respectively. The analysis also showed that high BMI (AOR: 2.19, 95 % C.I: 1.87–2.57) and having diabetes (AOR: 1.54, 95 % C.I: 1.31–1.83) were associated with the increasing risk of hypertension.

**Conclusions:**

Our study shows that the risk of hypertension was significantly associated with older age, sex, education, place of residence, working status, wealth index, BMI, and diabetes. Moreover, hypertension is largely untreated, especially in rural settings. The health system needs to develop appropriate strategies including early diagnosis, awareness via mass media, and health education programs for changing lifestyles should be initiated for older age, wealthy, and/or higher educated individuals in Bangladesh. Moreover, area-specific longitudinal research is necessary to find out the underlying causes of regional variations.

## Background

Hypertension is one of the major non-communicable diseases (NCDs) in the world, which significantly contributes to the burden of cardiovascular diseases (CVDs), stroke, kidney failure, disability, and premature death [1–3]. It is also identified as a global disease burden and is ranked third as a cause of disability-adjusted life-years (DALYs) [4]. According to the World Health Organization (WHO), about 17 million deaths occur worldwide due to CVDs, of which hypertension alone accounts for 9.4 million deaths [5, 6], and 80 % of the CVD-related deaths occurred in the developing countries [7]. The global prevalence of hypertension is projected to increase from 26 % in 2000 to 29.2 % by 2025 [5], which will be approximately 29 % of the world’s population. Although hypertension is more prevalent in developed counties like USA [8], its prevalence is increasing in the low and middle-income countries (LMIC) [1]. Countries in Asia, especially Southeast Asia, are having an increasing burden of hypertension including CVDs [9–11]. According to the WHO, hypertension has become a significant health concern in the Asian region, affecting more than 35 % of the adult population [12]. The two fast-growing economies, India, and China, have a huge burden of hypertension and are projected to proliferate by 2025 [13]. Bangladesh, a developing country in South Asia, has been experiencing an epidemiologic transition from communicable diseases to NCDs [14]. In recent years, rapid urbanization, increased life expectancy, unhealthy diet, and lifestyle changes have led to an increase in the rate of CVD including hypertension in Bangladesh [15]. The prevalence of hypertension was first reported as 1.10 % in 1976 in Bangladesh [16]. A systematic review and meta-analysis of the prevalence of hypertension in the country among 6,430 adults for the period 1995 to 2009 was estimated to be 13.5 % with a 95 % confidence interval (CI) ranging from 12.7–14.2 % [17]. Another meta-analysis for the prevalence of CVDs and type 2 diabetes between 1995 to 2010 found the pooled prevalence of hypertension to be 13.7 % (CI:12.1 %–15.3 %) [18]. Moreover, there was a wide range of variation in the prevalence of hypertension reported by several studies ranging from 11 to 44 % [17–21]. Due to the lack of representative data, [14, 17, 22] these studies were small-scale, confined to urban - rural communities or some other specific groups (e.g. slum residents), which cannot provide sufficient information for Bangladesh at large [14, 16, 23–25]. Also, a substantial proportion of the population with hypertension remains undiagnosed and not treated properly due to lack of access to health care and high treatment costs. Thus, this study was intended to assess the factors associated with hypertension in the general adult population in Bangladesh.

## Methods

### Study population

Bangladesh, located in the northeastern part of South Asia is one of the most densely populated countries in the world (1,015 people per sqkm), with a population of nearly 149.8 million in 2011 [26]. For administrative purposes, the country has divided into seven regions: south (Barishal), southeast (Chittagong), central (Dhaka), west (Khulna), midwestern corner (Rajshahi), northwest (Rangpur), and east (Sylhet). Including the capital in Dhaka, these administrative regions possess different demographic, environmental, and economic structures [26, 27]. All household members of age 35 years and older from both rural and urban areas were eligible to participate in the study.

### Data source

The Demographic Health Survey (DHS) was designed to collect data to monitor and evaluate population health and nutritional status of developing countries. In Bangladesh, this survey has been carried out continuously in three-year intervals since 1993 under the authority of the National Institute for Population Research and Training (NIPORT) of the Ministry of Health and Family Welfare. The data files are available at the DHS Program website [28]. The 2011 DHS was the first national survey in Bangladesh to incorporate the measurements of biomarker information including blood pressure and blood glucose measurements. For this study, we used the most recent available data of the 2011 Bangladesh Demographic and Health Survey (BDHS) including participants of age 35 years and older.

### Sampling design and sample size

**Fig. 1:**
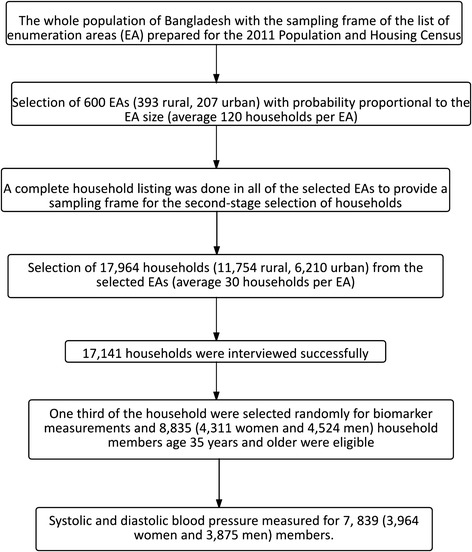
Sample method and sample size

The 2011 BDHS used two-stage stratified cluster sampling from non-institutionalized households [27]. The sampling frame used for the survey was the complete list of enumeration areas (EA) covering the whole country of the most recent population census prepared by Bangladesh Bureau of Statistics (BBS) [26]. An EA is a geographic area covering on average 113 households. In the first stage, 600 EAs (207 urban, 393 rural) were selected with probability proportional to the EA size. In the second stage, a systematic sample of 30 households on average was selected with an equal probability from each EA to provide statistically reliable estimates of key demographic and health variables for the country as a whole, for urban and rural areas separately, and for each of the seven divisions. With this design, the survey selected 17,964 (11,754 Rural, 6,210 Urban) residential households. Among the selected households, 17,141 households were interviewed successfully [27]. For measuring biomarker information, a subsample (one in three of the 17,511 eligible households) was selected. In this subsample, all women and men aged 35 years and older were eligible to participate in the biomarker component, which included blood pressure measurements, testing for anemia, blood glucose testing, and height and weight measurements. This subsample included a total of 8,835 (4,524 men and 4,311 women) household members age 35 years and older from 83,731 household members [27]. Among these individuals, 92 % of women and 86 % of men participated in the blood pressure measurement, and 89 % of women and 83 % of men participated in the blood glucose measurement. After excluding missing data and non-responses, the final sample was 7,839. The sample design and sample selection process is presented in Fig. 1. The detailed survey procedure, study method, and questionnaires are available in the final report of the 2011 BDHS [27].

### Ethical issues and consent

The 2011 BDHS received ethical approval from ICF Macro Institutional Review Board, Maryland, USA and National Research Ethics Committee of Bangladesh Medical Research Council (BMRC), Dhaka, Bangladesh. Informed consent was obtained from each participant of the survey before enrolling in the survey by using the Introduction and Consent form of the survey. It was also explained that the information will be kept strictly confidential and will not be shared with anyone except members of the survey team. All of these information were de-identified before the analyses.

### Outcome of interest

The outcome variable was having hypertension. The 2011 BDHS used the American Heart Association (AHA) guidelines for cut-off points for blood pressure measurements [29]. An individual was considered to have hypertension if systolic blood pressure (SBP) ≥ 140 mmHg (millimeters of mercury) and/or, diastolic blood pressure (DBP) ≥ 90 mmHg and/or taking anti-hypertensive medication during the survey. Pre-hypertension was defined by SBP ≥120 mmHg but < 140 mmHg and/or DBP ≥ 80 mmHg but < 90 mmHg and/or not taking anti-hypertensive medication at the time of survey [29]. Individuals with SBP ≤ 120 or DBP <80 mmHg or not taking anti-hypertensive medication were considered as non-hypertension [29]. For analytical purposes, we merged two groups (pre-hypertension and non-hypertension) together to make the variable dichotomous (person with hypertension versus no hypertension).

### Risk factors

The following variables were used as risk factors: age, sex, marital status, working status, wealth index, place of residence, region of residence, body mass index, and diabetes. The wealth index was calculated by the BDHS using the principal component analysis ranging from poorest to richest levels [27]. The body mass index was categorized into two categories: normal (BMI ≤24.99), overweight and obese (BMI ≥30). Due to fewer frequencies, underweight and overweight BMI categories were re-coded. The re-coded BMI variable considered underweight with normal and overweight with obese categories. Diabetes was defined as having a fasting plasma glucose level ≥ 7.0 mmol/L and/or taking diabetes medication at the time of the survey. Any person having either fasting plasma glucose (mmol/ L) level between 6.0 to 6.9 and no diabetes medication at the time of the survey was defined as a pre-diabetic person. Similarly, any person having either fasting plasma glucose level below 6.0 mmol/L and not taking diabetes medication was defined as a diabetes free person [30]. For analytical purposes, we merged two groups (pre-diabetes and diabetes free) together to make the variable dichotomous.

### Measurement of the disease

The 2011 BDHS used the LIFE SOURCE® UA-767 Plus Blood Pressure Monitor model; the automatic device included separate cuffs for measuring blood pressure in respondents with small, medium, and large arm circumferences. This model is one of the blood pressure monitors recommended for use by WHO. During the survey, blood pressure was measured and recorded by trained health technicians. Three measurements of both systolic and diastolic blood pressure were taken during the survey at approximately 10-minute intervals between measurements. The average of the second and third measurements was used to report respondents’ blood pressure values [27].

### Statistical analyses

A series of statistical analyses, such as multivariable logistic regression, has been performed. Descriptive information for the selected variables was provided first. Then cross-tabulations (i.e. bivariate) were performed to compare the hypertension status across covariate categories. A chi-square test was performed to assess the proportional differences in hypertension status across the selected categorical variables. Bivariate and multivariable logistic regression models were used to identify the significant risk factors for hypertension. Initially, potential risk factors were assessed using bivariate logistic regression analysis; an arbitrary p - value of < 0.20 was used as criteria to include it in the multivariable logistic regression model to control confounding effects, and the results were statistically significant at p- value of ≤ 0.05. For the multivariable logistic regression models, we calculated the crude odds ratios (COR) and adjusted odds ratio (AOR) with 95 % confidence interval (CI) for each independent variable. Appropriate sample weights provided by the 2011 BDHS were used for analysis. All statistical procedures were performed using the Statistical Analysis System (SAS) 9.4 for Windows (SAS Institute Inc., Cary, NC, USA).

## Results

**Fig. 2:**
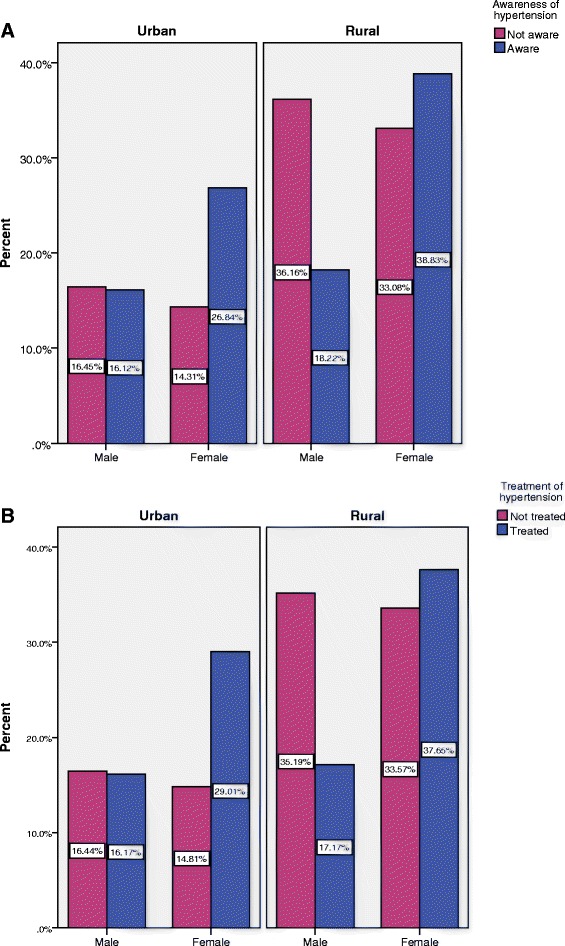
Awareness and treatment status of hypertension by sex and by place of residence, Bangladesh Demographic and Health Survey (BDHS), 2011

A summary of the socioeconomic, demographic, and anthropometric characteristics of the hypertensive and non- hypertensive study participants is presented in Table 1. Among the 7,839 subjects, 49.4 % were male and 50.6 % were female. The median age (±SD) of the study participants was 49.0 (±13) years. The mean SBP (±SD) and DBP (±SD) was 118.95 (21.36) and 78.03 (11.89), respectively. It was observed that study participants with older age (60–69 years), higher education, and higher wealth status had higher percentage of having hypertension compared to the study participants with a younger age (35–39 years), no education, and poor wealth status, respectively. The majority of the hypertensive respondents came from the richest households (30.3 %) followed by richer households (22.2 %), and other wealth index categories had a similar proportion of having hypertension (around 14 %). Hypertension was significantly associated with high BMI and having diabetes. Moreover, hypertension significantly varied by geographic region, marital status, and employment status. The distribution of level of education, wealth status, body mass index (BMI), and diabetes status by place of residence is shown in Table 2. It is found that urban study participants had higher educational attainment (12.5 % vs. 3.4 %) and higher wealth status (50.7 % vs. 10.8 %) compared to rural study participants. Similar proportion was also found in BMI and diabetes status of the respondents: urban individuals had higher BMI (25.7 %) and a higher proportion of diabetes (16 %). Figure 2 shows the awareness and treatment status of the hypertensive respondents by place of residence and gender. It is found that awareness and treatment among urban males are more or less similar whereas, rural men and women are less aware of their status of hypertension, and they are less likely to take medication to lower the blood pressure. Table 3 shows the risk factors associated with hypertension from the multivariable logistic regression analysis, together with adjusted and crude odds ratios, and 95 % confidence intervals after adjusting for a number of important covariates. Study participants with older age, female gender, higher education, higher socioeconomic status, living in urban areas, overweight and obesity, and diabetes, were more likely to have hypertension. The risk of hypertension was significantly higher among the individuals aged 60 to 69 years (AOR = 3.77, 95 % CI: 3.01–4.72) and those aged 70 years and older (AOR = 4.17, 95 % CI: 3.24–5.36) compared to the individuals aged 35–39 years old. In addition, respondents with higher education had 63 % (AOR: 1.63, 95 % CI: 1.24–2.13) of a higher chance of having hypertension compared to the respondents with no education. The multivariable logistic regression also indicates that the richest individuals were more likely to have hypertension compared to the poorest individuals (AOR: 1.91 (95 % CI1.54–2.38). The urban study participants were 16 % (AOR: 1.16, 95 % C.I: 1.00–1.35) more likely to have hypertension compared to the rural respondents. The risk of hypertension was found to be significantly lower (AOR: 0.64, CI: 0.53–0.77) among employed individuals compared to those with no employment. There was a noticeable variation in risk of hypertension among seven administrative divisions of Bangladesh: the risk was 46 % higher among the individuals from Rangpur (AOR: 1.46, CI: 1.09–1.94) and 35 % higher among the individuals from Khulna (AOR: 1.35, 95 % CI: 1.02–1.79) division compared to the individuals from Barisal division. The odds of having hypertension among overweight and obese respondents was found to be 2.19 (95 % CI: 1.87–2.57) times higher compared to normal-weight respondents. For individuals with diabetes, the odds of having hypertension were 54 % (AOR: 1.54, 95 % CI: 1.31–1.83) higher than the individuals without diabetes.

**Table 1:** Characteristic of the study participants by hypertension status, Bangladesh Demographic and Health Survey (BDHS), 2011

	Total		No hypertension		Hypertension		
Variables	n	%	n	%	n	%	
Age group							^***^
35–39	1477	18.8	1267	21.8	210	10.4	
40–44	1372	17.5	1096	18.8	276	13.7	
45–49	1213	15.5	923	15.8	290	14.4	
50–54	1053	13.4	777	13.3	276	13.7	
55–59	689	8.8	482	8.3	207	10.3	
60–69	1095	14.0	710	12.2	385	19.1	
70+	940	12.0	569	9.8	371	18.4	
Sex							^***^
Male	3875	49.4	3122	53.6	753	37.4	
Female	3964	50.6	2701	46.4	1263	62.6	
Marital status							^***^
Currently married	6595	84.1	5091	87.4	1504	74.6	
Others	1243	15.9	732	12.6	511	25.4	
Education							^**^
No education	3724	47.5	2732	46.9	992	49.2	
Primary education	2560	32.7	1954	33.6	606	30.1	
Secondary education	1108	14.1	816	14.0	292	14.5	
Higher education	446	5.7	321	5.5	125	6.2	
Working status							^***^
No	4073	52.0	2723	46.8	1350	67.0	
Yes	3762	48.0	3098	53.2	664	33.0	
Wealth index							^***^
Poorest	1524	19.4	1237	21.2	287	14.2	
Poorer	1508	19.2	1182	20.3	326	16.2	
Middle	1551	19.8	1205	20.7	346	17.2	
Richer	1619	20.7	1172	20.1	447	22.2	
Richest	1637	20.9	1027	17.6	610	30.3	
Place of residence							^***^
Rural	6009	76.7	4590	78.8	1419	70.4	
Urban	1829	23.3	1233	21.2	596	29.6	
Division of residence							^***^
Barisal	464	5.9	350	6.0	114	5.7	
Chittagong	1334	17	1041	17.9	293	14.5	
Dhaka	2514	32.1	1834	31.5	680	33.7	
Khulna	1020	13	712	12.2	308	15.3	
Rajshahi	1136	14.5	867	14.9	269	13.3	
Rangpur	922	11.8	663	11.4	259	12.9	
Sylhet	449	5.7	357	6.1	92	4.6	
Body mass index							^***^
Normal	6586	86.5	5088	89.8	1480	76.9	
Overweight or obese	1023	13.5	579	10.2	444	23.1	
Diabetes status							^***^
No	6699	89.0	5074	90.9	1625	83.6	
Yes	825	11.0	506	9.1	319	16.4	

Notes: ^***^ < 0.01, ^**^ < 0.5, ^*^ < 0.1

**Table 2:** Distribution of education, wealth status, body mass index (BMI), and diabetes status by place of residence, Bangladesh Demographic and Health Survey (BDHS), 2011

	Total		Urban		Rural	
Variables	n	%	n	%	n	%
Education						
No education	3537	44.8	867	33.5	2670	50.4
Primary education	2656	33.7	860	33.2	1796	33.9
Secondary education	1192	15.1	539	20.8	653	12.3
Higher education	502	6.4	323	12.5	179	3.4
Wealth index						
Poorest	1403	17.8	199	7.7	1204	22.7
Poorer	1428	18.1	169	6.5	1259	23.8
Middle	1529	19.4	292	11.3	1237	23.3
Richer	1642	20.8	617	23.8	1025	19.3
Richest	1885	23.9	1312	50.7	573	10.8
Body mass index						
Normal	6498	85.1	1864	74.3	4634	90.3
Overweight or obese	1142	14.9	644	25.7	498	9.7
Diabetes status						
No	6663	88.3	2084	84	4579	90.5
Yes	879	11.7	397	16	482	9.5

**Table 3:** Bivariate and multivariable logistic regression analysis of factors associated with hypertension among adults in Bangladesh, Bangladesh Demographic and Health Survey (BDHS), 2011

Variables	COR	CI		AOR	CI	
Age group						
35–39	Ref			Ref		
40–44	1.43	1.17–1.75	^**^	1.45	1.18–1.79	^**^
45–49	1.88	1.54–2.30	^***^	2.00	1.62–2.47	^***^
50–54	2.00	1.63–2.46	^***^	2.56	2.05–3.20	^***^
55–59	2.51	2.01–3.14	^***^	2.71	2.12–3.46	^***^
60–69	3.09	2.54–3.76	^***^	3.77	3.01–4.72	^***^
70+	3.59	2.93–4.40	^***^	4.16	3.24–5.36	^***^
Sex						
Male	Ref			Ref		
Female	1.93	1.74–2.15	^***^	1.44	1.19–1.74	^**^
Marital status						
Currently married	Ref			Ref		
Others	2.29	2.00–2.62	^***^	1.36	1.15–1.60	^***^
Education						
No education	Ref			Ref		
Primary education	1.16	1.03–1.31	^**^	1.11	0.97–1.28	
Secondary education	1.17	0.99–1.38	^*^	1.35	1.12–1.63	^**^
Higher education	1.27	1.00–1.60	^**^	1.63	1.25–2.14	^***^
Working status						
No	Ref			Ref		
Yes	0.43	0.39–0.49	^***^	0.64	0.53–0.77	^***^
Wealth index						
Poorest	Ref			Ref		
Poorer	1.21	1.01–1.46	^**^	1.22	1.01–1.48	^**^
Middle	1.28	1.07–1.54	^**^	1.21	1.00–1.48	^**^
Richer	1.69	1.42–2.01	^***^	1.50	1.23–1.82	^***^
Richest	2.61	2.20–3.09	^***^	1.91	1.54–2.38	^***^
Place of residence						
Rural	Ref			Ref		
Urban	1.57	1.39–1.77	^***^	1.16	1.00–1.35	^*^
Division of residence						
Barisal	Ref			Ref		
Chittagong	0.90	0.69–1.17		0.72	0.54–0.96	^**^
Dhaka	1.16	0.91–1.48		1.04	0.84–1.36	
Khulna	1.33	1.02–1.74	^**^	1.35	1.02–1.79	^**^
Rajshahi	1.01	0.77–1.32		1.07	0.81–1.43	
Rangpur	1.23	0.93–1.60		1.46	1.09–1.94	^**^
Sylhet	0.82	0.59–1.13		0.75	0.52–1.06	^*^
Body mass index						
Normal	Ref			Ref		
Overweight or obese	2.65	2.30–3.04	^***^	2.19	1.87–2.57	^***^
Diabetes status						
No	Ref			Ref		
Yes	1.99	1.71–2.32	^***^	1.54	1.31–1.83	^***^

*COR* Crude odds ratio, *AOR* Adjusted odds ratio

Notes: ^***^ < 0.01, ^**^ < 0.5, ^*^ < 0.1

## Discussion

In this population-based, cross-sectional, and nationally representative study among the adults in Bangladesh, we found that older age, female, higher education, wealthier socioeconomic status, diabetes, and high BMI (overweight and obesity) were significant factors associated with hypertension, which is consistent with the previous studies in Bangladesh and other studies in developing countries [24, 31–37]. We also found that a significant proportion of rural adults was not aware of this disease. Study participants with age 60–69 years had higher odds of (AOR = 3.77) having hypertension than those of 35–39 years old. Moreover, we observed a significant positive association between increasing age and hypertension. Since age is an unmodifiable risk factor [38], in Bangladesh, the population age structure is changing due to the decline in fertility level and a steady increase in life expectancy. The number of old age population will increase rapidly, which will strengthen the risk of hypertension among older age population in the near future [39]. Therefore, other modifiable factors should be taken into consideration through intervention programs. For example, reducing weight and cutting fatty foods from daily meals could be an option. Moreover, respondent’s education and wealth status were positively associated with the risk of hypertension in our study. A similar positive correlation was also observed in the low and middle-income countries, whereas an inverse correlation was found in developed countries [24, 31]. More clearly, in low and middle income countries, individuals having higher education belong to the richest wealth quintile, which leads them to have luxurious lifestyles and consumption of more high-caloric foods. As a result, the body weight of individuals has increased, and their physical activity has decreased, which increase the likelihood of having hypertension [34, 40–44]. We found that the odds of having hypertension was higher among urban respondents, which is also consistent with other studies in Bangladesh [44] and India [45]. The main reason for this could be consumption of high junk food and less physical activity among the urban respondents [44, 46]. In addition, the rural people are more likely to be engaged in daily household and other labor-intensive activities that may keep them physically active, burn more calories and lead to lower BMI [47]. In our study, the highest risk (AOR: 1.47) of hypertension was observed in the northwestern part (Rangpur Division) of the country, whereas the lowest risk was found in the eastern part (Sylhet division). This variation could be influenced by more intake of raw salt, poverty, malnutrition, and dietary habits [48, 49]. Further study will be required to investigate the variation in different geographic regions of Bangladesh. We also found high BMI (overweight and obesity) and diabetes to be a significant factor associated with hypertension. Similar findings were found in a study in India and Bangladesh conducted by the WHO [50] and other recent studies [51]. Since the relationship between hypertension and BMI is well established [51], further study is needed to investigate the progression of BMI with diet and physical activity. Unplanned development in the urban areas has created an environment that is prohibitive and unsafe for physical activity [52]. Popularity of and increased access to fast food may also contribute to poorer diet quality, among the city’s affluent class [53]. Studies in some developed countries demonstrate that urbanization is one of the important factors of hypertension [54–56]; however, in our analysis, urbanization was not included due to limited data. Hypertension was found to be associated with diabetes. However, epidemiological studies and pathophysiological mechanism as well other studies in Bangladesh reported the coexistence of hypertension [39, 57].

### Strengths and limitations

The major strengths of our study are the use of a nationally representative survey data with comprehensive information on hypertension, using anthropometric and demographic variables. Moreover, the measurements of hypertension were collected by trained and experienced health technicians using WHO-recommended methods rather self-reporting. Since DHS uses standard and valid data collection tools, the measurement error and bias is less in this study compared to other cross-sectional studies in Bangladesh. In spite of some good strengths, our study also has some limitations. The main limitation was that some other important factors of hypertension like diet, physical exercise, family history of hypertension, HDL- cholesterol level, smoking status, salt intake, and impact of urbanization were not included in the analysis as they are not available in the 2011 BDHS data. Moreover, the 2011 BDHS is a cross-sectional survey, and an individual’s blood pressure was taken in the survey for one day only. Therefore, we do not have longitudinal data for the factors associated with hypertension. Additionally, we only considered individuals of age 35 years and older to provide the biomarker information. Therefore, results of this study may not be extended to the younger age groups.

## Conclusions

In this study, we found that there is a wide range of factors, which are significantly associated with hypertension among the adults (age ≥35 years) in Bangladesh. The findings demonstrate that individuals with older age, higher socioeconomic status, higher education, high BMI, and diabetes have a significant influence on the odds of having hypertension. Moreover, a significant proportion of the adult men and women are not aware of the consequence of this disease, and a small proportion of them are taking antihypertensive drugs. The implications of the findings are important since a large proportion of the population in Bangladesh are adults and if corrective actions are not taken, there will be unbearable health consequences. Since most of these factors associated with hypertension are modifiable and preventable, early diagnosis, preventive behavior, and taking policies can reduce the odds of having the disease. Therefore, the health system of the country needs to develop strategies to increase the required screening and diagnosis of the hypertension to both rural and urban areas. Comprehensive and integrated intervention programs should be implemented to make awareness so that the primary health care services go towards the primary prevention and management of the needs of older adults. These interventions may include changing lifestyle and food habits at the community level to reduce the future burden of the disease. Furthermore, longitudinal research is required to find out the underlying causes of risk of hypertension across the regions of Bangladesh.

## Abbreviations

AHA: American Heart Association
BBS: Bangladesh Bureau of Statistics
BMI: Body mass index
BMRC: Bangladesh Medical and Research Council
CI: Confidence interval
COR: Crude odds ratios
CVD: Cardiovascular disease
DALY: Disability adjusted life years
DHS: Demographic Health Survey
DBP: Diastolic blood pressure
EA: Enumeration area
ICF International: A Technology, Policy, and Management consulting services company
LMIC: Low and middle-income countries
NCD: Non-communicable diseases
NIPORT: National Institute for Population Research and Training
AOR: Adjusted odds ratio
SBP: Systolic blood pressure
USAID: U.S. Agency for International Development
WHO: World Health Organization
